# Paintball Purpura: The Ball Sports-induced Targetoid Erythema (SITE) Sign Revisited

**DOI:** 10.7759/cureus.8056

**Published:** 2020-05-11

**Authors:** Shahzeb Hassan, Philip R Cohen

**Affiliations:** 1 Dermatology, Northwestern University Feinberg School of Medicine, Chicago, USA; 2 Dermatology, San Diego Family Dermatology, National City, USA

**Keywords:** ball, ecchymoses, erythema, paint, paintball, purpura, sign, sports, targetoid

## Abstract

The ball sports-induced targetoid erythema (SITE) sign is a term that refers to annular erythematous lesions surrounding normal skin that occur after ball to skin contact. Associated purpura may be present or absent. A 19-year-old college student presented with multiple purpuric lesions of targetoid appearance. Additional history revealed that the lesions corresponded with the areas of ball contact during a paintball game. Similar lesions have been described in the participants of other sports, including floorball, ping pong, racquetball, and squash. When evaluating a patient with targetoid erythema with or without accompanying purpura, additional history of recent participation is an activity involving a high-velocity ball may be useful for establishing the diagnosis of the ball SITE sign.

## Introduction

Dermatoses may be associated with ball sports. One condition that has been observed is the ball sports-induced targetoid erythema (SITE) sign. The lesion may occur in participants of sports in which the size of the ball is usually small and the velocity is high [1].

The ball SITE sign was previously described as sports purpura [2]. However, this is a misnomer since the lesions can also be ecchymotic or both purpuric and ecchymotic [1]. A man who developed several purpuric targetoid lesions following contact from paintballs with his skin is described, and the features of the ball SITE sign are reviewed.

## Case presentation

**Figure 1:**
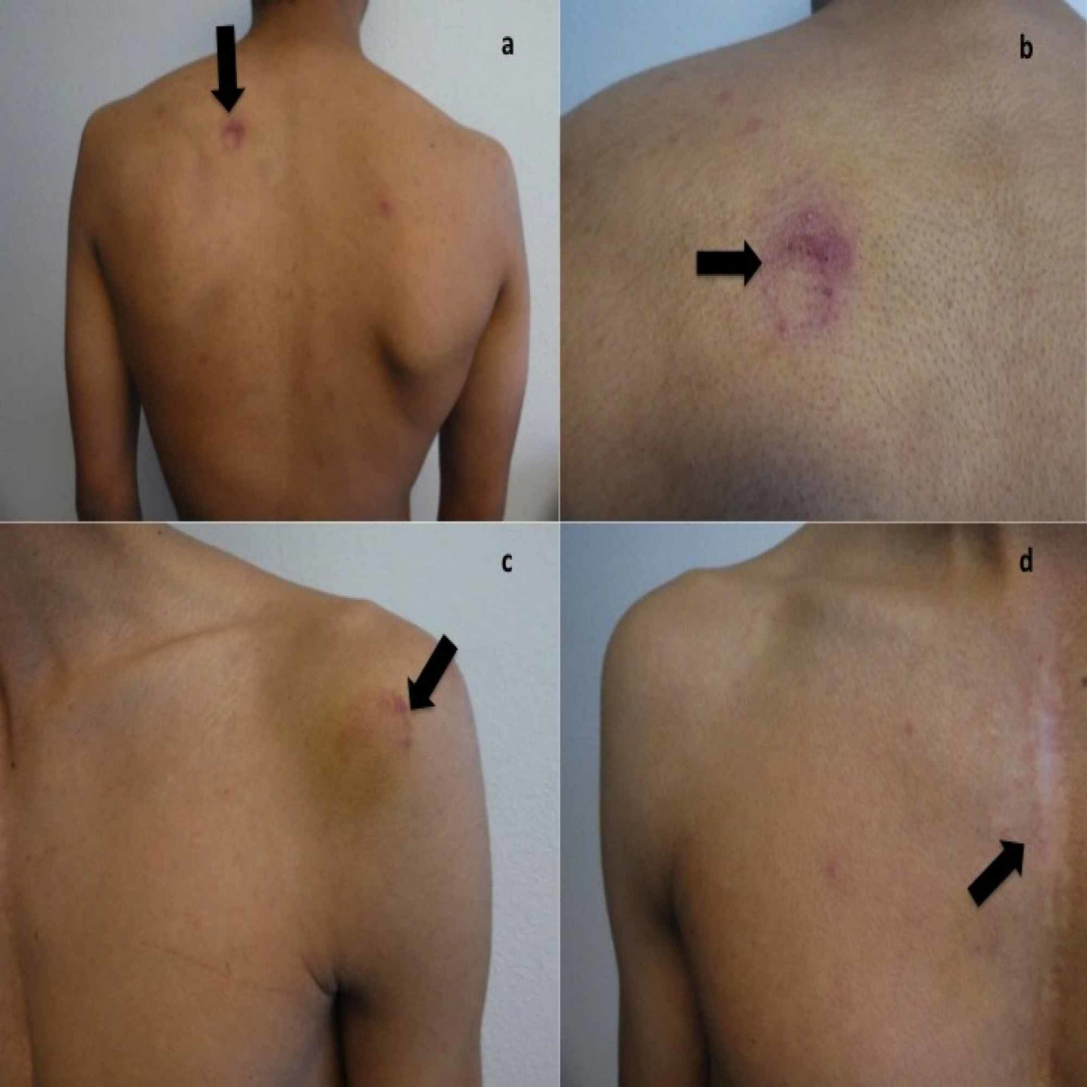
Paintball-associated ball sports-induced targetoid erythema (SITE) sign presenting as annular erythema on the left scapula, the left shoulder, and the left anterior chest. Distant (a) and closer (b) views of the left scapula of a 14-year-old man show annular ecchymosis at the site of contact with a paintball (black arrows) three days earlier. The left shoulder (c) and left anterior chest (d) also show annular ecchymosis at the site of contact with a paintball (black arrows) that occurred three days earlier.

A 19-year-old college student presented for evaluation of multiple asymptomatic red, skin lesions. Cutaneous examination showed three purpuric lesions that had a targetoid appearance of ecchymosis with normal-appearing skin in the center on his left scapula, left anterior shoulder, and left chest (Figure 1).

Additional history revealed that the patient had participated in a paintball activity three days earlier. The location of his cutaneous lesions corresponded to the sites where the paintball had contacted his skin. Correlation of the history and clinical presentation established the diagnosis of paintball-associated ball SITE sign.

Follow-up examination one week later (which was 10 days after the causative event) showed partial spontaneous resolution of the purpuric lesions on the left scapula and left anterior shoulder. The left chest lesion had completely resolved.

## Discussion

The ball SITE sign has been associated with various sports (Table 1) [1-10]. The features of the balls that can cause ball SITE sign vary. The composition can be gelatin (paintball), plastic (ping pong ball), or rubber (racquetball and squash ball) [1].

**Table 1:** Sports associated with the ball sports-induced targetoid erythema (SITE) sign ^a^Nomenclature of floorball-associated ball SITE sign includes floorball ecchymotic patches and floorball purpura. ^b^Nomenclature of paintball-associated ball SITE sign includes paintball purpura, paint pellet erythema, and paint pellet purpura. ^c^Nomenclature of ping pong-associated ball SITE sign includes ping pong patches. ^d^Nomenclature of racquetball-associated ball SITE sign includes annular erythematous (and occasionally purpuric) patches, annular lesion, and racquetball-associated targetoid erythema (RATE) sign. ^e^Nomenclature of squash-associated ball SITE sign includes annular erythematous (and occasionally purpuric) patches, annular lesion, and targetoid erythema associated with squash (TEAS) sign.

Sport	Reference
Floorball^a^	[1-3]
Paintball^b^	[4-8]
Ping pong^c^	[9]
Racquetball^d^	[1,8,10]
Squash^e^	[1,8,10]

The weight of the ball can be as low as 2.5 grams for ping pong balls and as high as 58.5 grams for racquetballs. The weight for squash balls can be as high as 25 grams. In contrast, paintballs weigh approximately 3.3 grams [1,5,6,9,11].

The diameters of the balls also vary. Racquetballs, squash balls, and ping pong balls all have greater diameters of approximately 57 millimeters, 40 millimeters, and 39 millimeters, respectively. In contrast, the paintball has a much smaller diameter of only 17 millimeters [1,5,6,9,11].

The speed of the ball is usually fast. Paintballs have been observed to travel at 191 to 205 miles per hour. In contrast, the other balls travel slower than the paintballs. Ping pong balls travel at 100 miles per hour, whereas racquetballs (traveling 77 to 191 miles per hour) and squash balls (traveling greater than 150 miles per hour) often move faster [1,5,6,9,11]. 

Our patient developed the ball SITE sign following contact with his skin from paintballs. The ball SITE sign occurring in participants receiving skin impact from paintballs has been described previously (Table 2) [4-8]. 

**Table 2:** Clinical characteristics of patients with paintball-associated ball sports-induced targeted erythema (SITE) sign. CR, case report; pub, publication; ref, references; SITE, sports-induced targetoid erythema.

Author, pub year	Comment	Ref
Siegel et al., 1980	First report of a patient with paint pellet purpura. A 32-year-old woman had two lesions measuring 8-10 cm with ecchymotic margins and clear centers on her arm and back. These were the sites where she was hit by paint bullets during a survival game two days prior to her office visit.	[8]
Rahbari and Nabai, 1996	A 19-year-old white man presented with three annular, erythematous lesions on his upper back. He mentioned that he had been hit by several paint pellets two days earlier.	[7]
Metelitsa et al., 2004	The authors, in their review of sports-related dermatoses, mention their personal observations of paintball purpura presenting as a large purpuric patch at the location where the paintball fired from an air-powered gun impacted the skin.	[4]
Levsky and Crowe, 2005	A 25-year-old woman had a mildly tender, non-blanching, erythematous ring-shaped lesion on the left forearm. Other people who had been in contact with the patient also had similar lesions on their extremities and/or trunk.	[5]
Aboutalebi and Stetson, 2005	A 14-year-old man and his 12-year-old brother presented with multiple lesions on the trunk following a paintball game on the prior weekend. The lesions appeared as annular purpuric patches located at the sites struck by the paintballs.	[6]
Hassan and Cohen, 2020	Comprehensive review of paintball associated ball SITE sign. A 19-year-old man with cutaneous lesions caused by impact with paintballs that occurred three days prior to his office visit.	

What is now referred to as paintball purpura was initially described as paint pellet purpura by Siegel in 1986 [8]. Subsequently, ball SITE sign associated with paintballs has been described as paintball purpura and paint pellet erythema. The patients ranged in age from 14 to 32 years [4-8]. The morphology of their lesions was either erythema, purpura, or both [4-8].

The differential diagnosis of ball SITE sign is described in Table 3 [12-16]. Although there are several clinical lesions that can mimic the morphology of the ball SITE sign, the associated history of trauma to the skin site from a high-velocity ball is helpful in establishing the diagnosis. Therefore, a biopsy is usually not necessary to confirm the diagnosis of the ball SITE sign.

**Table 3:** Differential diagnosis of ball sports-induced targetoid erythema (SITE) sign ^a^These include cupping, factitial dermatitis, and physical abuse. ^b^This includes tinea corporis. ^c^These include dermatitis medicamentosa and fixed drug eruption. ^d^These include granuloma annulare, insect bite reaction, Majocchi’s disease (purpura annularis telangiectodes), and subacute cutaneous lupus erythematosus. ^e^These include erythema annulare centrifugum, erythema chronicum migrans, erythema multiforme, gyrate erythemas, and urticaria.

Differential diagnosis	Reference
Exogenous^a^	[12]
Infectious^b ^	[13]
Medication related^c^	[14]
Miscellaneous^d^	[15]
Reactive erythema^e ^	[13,16]

The treatment of paintball purpura is symptomatic. They lesions typically resolve spontaneously as they did in our patient. Also, they often reoccur on new sites of skin contact when the individual again participates in the activity. 

## Conclusions

The term ball SITE sign was introduced to characterize lesions of targetoid erythema that present after high-velocity contact of ball to skin; these lesions occur in the setting of various sports, including paintball, ping pong, racquetball, and squash. The annular red ring of erythema may or may not concurrently present with purpura; our patient’s lesions had purpura. Also, similar to our patient, the lesions typically resolve spontaneously; therefore, a proper history is necessary to establish the diagnosis and avoid additional evaluation or unnecessary treatment.

## References

[1] Cohen PR (2015). The ball SITE sign: ball sports-induced targetoid erythema in a racquetball player. Dermatol Pract Concept.

[2] Kluger N (2015). Sports purpura from floorball, indoor climbing, and archery. Cutis.

[3] Kluger N (2012). Purpura du sportif. (Article in French). Presse Med.

[4] Metelitsa A, Barankin B, Lin AN (2004). Diagnosis of sports-related dermatoses. Int J Dermatol.

[5] Levsky ME, Crowe M (2005). What is your diagnosis? Paintball purpura. Cutis.

[6] Aboutalebi S, Stetson CL (2005). Paintball purpura. J Am Acad Dermatol.

[7] Rahbari H, Nabai H (1996). Paint pellet erythema. Pediatr Dermatol.

[8] Siegel DM, Goldberg LH, Altman AR, Kalter DC (1986). Paint pellet purpura: a peril for pistol-packing paramilitary personnel. JAMA.

[9] Scott MJ Jr, Scott MJ 3rd (1989). Ping pong patches. Cutis.

[10] Barazi H, Adams BB (2006). Sports purpura. Int J Dermatol.

[11] Conn JM, Annest JL, Gilchrist J, Ryan GW (2004). Injuries from paintball game related activities in the United States, 1997-2001. Inj Prev.

[12] Saha A, Seth J, Gorai S, Bindal A (2015). Dermatitis artefacta: a review of five cases: a diagnostic and therapeutic challenge. Indian J Dermatol.

[13] Trayes KP, Savage K, Studdiford JS (2018). Annular lesions: diagnosis and treatment. Am Fam Physician.

[14] Cohen PR (2017). Fixed drug eruption to supplement containing ginkgo biloba and vinpocetine: a case report and review of related cutaneous side effects. J Clin Aesthet Dermatol.

[15] Bernacchi E, Neri R, Caproni M, Loggini B, Fabbri P, Bombardieri S (2013). Annular subacute cutaneous lupus erythematosus lesions and polymyositis onset in a patient with primary Sjögren’s syndrome: how should this unusual association be classified?. Lupus.

[16] Tyring SK (1993). Reactive erythemas: erythema annulare centrifugum and erythema gyratum repens. Clin Dermatol.

